# Evaluation of Analgesic and Anti-Inflammatory Activities of Water Extract of* Galla Chinensis In Vivo* Models

**DOI:** 10.1155/2018/6784032

**Published:** 2018-02-18

**Authors:** Kai Sun, Xu Song, RenYong Jia, Zhongqiong Yin, Yuanfeng Zou, Lixia Li, Lizi Yin, Changliang He, Xiaoxia Liang, Guizhou Yue, Qiankun Cui, Yi Yang

**Affiliations:** ^1^Natural Medicine Research Center, College of Veterinary Medicine, Sichuan Agricultural University, Chengdu 611130, China; ^2^Key Laboratory of Animal Disease and Human Health of Sichuan Province, Sichuan Agricultural University, Chengdu 611130, China; ^3^College of Science, Sichuan Agricultural University, Ya'an 625014, China

## Abstract

**Aim:**

Pain and inflammation are associated with many diseases in humans and animals.* Galla Chinensis*, a traditional Chinese medicine, has a variety of pharmacological properties. The purpose of this study was to evaluate analgesic and anti-inflammatory activities of* Galla Chinensis *through different animal models.

**Method:**

The analgesic activities were evaluated by hot-plate and writhing tests. The anti-inflammatory effects were assessed by ear edema, capillary permeability, and paw edema tests. The contents of cytokines (NO, iNOS, PGE_2_, and IL-10) in serum of rats in paw edema test were inspected by ELISA assays.

**Results:**

In the hot-plate test,* Galla Chinensis* could significantly extend pain threshold when compared to control group. The inhibitory rates of writhes ranged from 36.62% to 68.57% in* Galla Chinensis*-treated mice. Treatment with* Galla Chinensis* (1 and 0.5 g/kg) could significantly inhibit ear edema (47.45 and 36.91%, resp.; *P* < 0.01).* Galla Chinensis* (1 g/kg) had significant (*P* < 0.05) anti-inflammatory activity in capillary permeability test (29.04%). In carrageenan-induced edema test, the inhibitory rates were 43.71% and 44.07% (*P* < 0.01) at 1 h and 2 h after administration of* Galla Chinensis *(1 g/kg), respectively, and the levels of proinflammatory cytokines were significantly reduced.

**Conclusion:**

These results suggest that* Galla Chinensis* has analgesic and anti-inflammatory effects, which may be a candidate drug for the treatment of inflammation and pain.

## 1. Introduction

Recent studies revealed that inflammation was associated not only with ailments, wounds, trauma, and swelling [1], but also with cancer, atherosclerosis, cardiovascular disease, arthritis, neurodegenerative disease, diabetes mellitus, and obesity [2]. Some physical, chemical, and biological stimuli can lead to occurrence of inflammation. It is usually accompanied with pain, redness, swelling, heat, and dysfunction [3]. The primary treatment of inflammation and pain is to use nonsteroidal anti-inflammatory drugs, but long-term use could lead to a lot of side effects, such as cardiovascular and gastrointestinal complications [4, 5]. Therefore, it is necessary to develop new drugs for treatment of inflammation and pain.

*Galla Chinensis*, a traditional Chinese herbal medicine, is widely distributed in Guizhou, Sichuan, Hubei, Hunan, Shanxi, and Yunnan. It originates from the plants of family* Anacardiaceae* (mainly* Rhus chinensis *Mill,* Rhus potaninii *Maxim, and* Rhus punjabensis*) parasitized by the Chinese sumac aphid Baker [6, 7]. The main ingredient is tannin acid.* In vitro* and* in vivo* studies have demonstrated its pharmacological effects, including spermicidal, antibacterial, and antioxidant activities [8, 9]. However, there are no reports about the analgesic and anti-inflammatory activities. Therefore, in this study, the analgesic effects of* Galla Chinensis* were determined through hot-plate test and acetic acid-induced writhing, and the anti-inflammatory activities were assessed through xylene-induced ear edema test, acetic acid-induced capillary permeability, and carrageenin-induced paw edema test for developing a new drug to treat inflammation and pain.

## 2. Materials and Methods

### 2.1. Animals

Male Sprague-Dawley rats (200 ± 20 g) and male or female Kunming mice (20 ± 2 g) were purchased from Chengdu Dossy Experimental Animals Co. Ltd. (License number SCXK (Sichuan) 201509). The experimental protocol was approved by the National Institute of Ethics Committee at Sichuan Agricultural University [approval number SYXK (Sichuan) 2014-187]. All animals were maintained in animal room with free access to water and standard diet and 12 h light-dark cycle. They were acclimatized at least 1 week before experiments started.

### 2.2. Drug and Chemicals

*Galla Chinensis* (number 160512) was bought from Baoji F. S. Biological Development Co. Ltd. (Shanxi, China) and identified by Dr. Lixia Li (Sichuan Agricultural University, China). Nitrogen monoxide (NO), nitric oxide synthase (iNOS), prostaglandin E_2_ (PGE_2_), and interleukin-10 (IL-10) were all bought from Nanjing Jiancheng Biotechnology Co., Ltd. (Nanjing, China).

#### 2.2.1. Extraction

*Galla Chinensis* powder (100 g) was decocted in water (1500 mL) for 2 h, and then the decoction was concentrated under reduced pressure. Finally, the* Galla Chinensis* extract (gallotannins) was obtained by lyophilization [10–12]. Referring to the method in the Chinese Pharmacopoeia, tannin of crude extract was determined by a tungsten molybdophosphate-casein colorimetric method. The results show that the content of tannin in crude extract is 92.5% [13].

*Galla Chinensis* extract (gallotannins) was dissolved in distilled water and administered orally. Indomethacin (positive control) was purchased from Shanxi Taiyuan Pharmaceutical Co. Ltd. (Shanxi, China) and dissolved in 0.5% (w/v) sodium carboxymethyl cellulose.

### 2.3. Hot-Plate Test

The hot-plate test was conducted as the methods previously reported [14, 15]. The temperature of electrical hot-plate was set at 55 ± 0.5°C. The female mice were put on electrical hot-plate, and the time was recorded when the mice showed any nociceptive indicators. The pain indicators consisted of lifting, licking paw, and jumping. A pain threshold of the female mice (the time when they first exhibit one of the pain indicators) within 5~30 s was used for the test. The mice were treated once a day with physiological saline,* Galla Chinensis *(1, 0.5, and 0.25 g/kg) and indomethacin (2 mg/kg) for 4 days, respectively. The pain threshold was measured at 30, 60, 90, and 120 min after the last administration, respectively. If the times were more than 60 s, the mice were taken out immediately and the response time was recorded as 60 s.

### 2.4. Acetic Acid-Induced Writhing in Mice

The acetic acid-induced writhing test was performed as previously described [16]. Mice were divided into five groups, and each group contained five male and five female mice. The mice received physiological saline, indomethacin (2 mg/kg), and* Galla Chinensis* at doses of 1, 0.5, and 0.25 g/kg for four consecutive days, respectively. The mice were intraperitoneally infected with 0.7% (v/v) acetic acid (0.01 mL/g) at 1 h after the last administration. The mice were then placed in an observation box, and the total number of writhes was recorded for 20 min after injection. The percentage of analgesia was calculated by the following equation:

(1) $$\begin{array}{c} \%\;\;Inhibition=\frac{N_{m}-N_{c}}{N_{m}}\times 100, \end{array}$$

where *N*_*m*_ is the number of writhes in control group; *N*_*c*_ is the number of writhes in treated groups.

### 2.5. Xylene-Induced Ear Edema

Mice were divided into five groups, and each group contained five male and five female mice. In control group, the mice received physiological saline (0.1 mL/10 g); indomethacin (2 mg/kg) was served as positive control;* Galla Chinensis* was administrated at doses of 1 g/kg, 0.5 g/kg, and 0.25 g/kg, respectively. The doses were administered orally to the mice each day for 4 consecutive days, respectively. At 1 h after the last administration, xylene (50 uL) was smeared on the surface of right ear [17]. The left ear was used as control. All animals were sacrificed at 1 h after smear, and then two ears along the auricle were cut off. The auricle with diameter of 8 mm was taken and weighed. The inhibition rate was calculated according to the following formula:

(2) %  Edema  degree=Mright−Mleft,%  Inhibition=Edema  degreecontrol−Edema  degreetreatedEdema  degreecontrol×100,

where *M*_right_ is the weight of right piece and *M*_left_ is the weight of left piece.

### 2.6. Acetic Acid-Indeed Capillary Permeability

Mice were divided into five groups, and each had ten mice (5 female and 5 male). The mice were given physiological saline, indomethacin (positive control; 2 mg/kg), and* Galla Chinensis* (1, 0.5, and 0.25 g/kg) once a day for 4 days, respectively. Evans blue (0.5%) was injected into tail vein at 1 h after last administration. The mice were intraperitoneally injected with 0.2 mL 0.7% (v/v) acetic acid. After 20 min, the pleural cavity was repeatedly washed with 6 ml saline, and then the liquid was collected and centrifuged at 3000 rpm for 10 min. The OD values at 590 nm of the supernatant were measured by a spectrophotometer [18].

### 2.7. Carrageenan-Induced Rat Paw Edema

The carrageenan-induced paw edema test was conducted according to the method previously reported [19]. Male rats were randomly divided into five groups (*n* = 8). The mice received physiological saline,* Galla Chinensis *(1, 0.5, and 0.25 g/kg), and indomethacin (positive control; 2 mg/kg), respectively, for 4 successive days. At 60 min after the last treatment, mice were infected with 0.1 mL of carrageenan suspension (0.1 g/mL) in the right hind paw. The swelling degree of paw was reflected by examining the average volume of paw swelling at 1, 2, 3, 4, 5, and 6 h, respectively. The edema degree was calculated according to the following formula:

(3) %  Inhibition=V1−V2V2×100,%  Inhibition=Edema  degreecontrol−Edema  degreetreatedEdema  degreecontrol×100,

where *V*_1_ is the average volume of paw swelling at different time per group. *V*_2_ is the average volume of paw before injecting carrageenan per group.

After the last measurement, all rats were anesthetized with ether, and the blood was gathered in vacuum tubes with no heparin sodium from abdominal aorta. After coagulation, the blood was centrifuged at 3000 rpm for 10 min and serum was collected. The contents of cytokines in serum were inspected by the ELISA assays, including NO, iNOS, PGE_2_, and IL-10.

### 2.8. Statistical Analysis

All statistical analysis was determined using SPSS 19.0 statistical software and one-way analysis of variance (ANOVA) in Duncan's test. *P* < 0.05 was regarded as statistically significant and *P* < 0.01 was considered very significant. The data were expressed as mean value ± standard deviation (M ± SD).

## 3. Result

### 3.1. Electrical Hot-Plate Study in Mice

**Figure 1:**
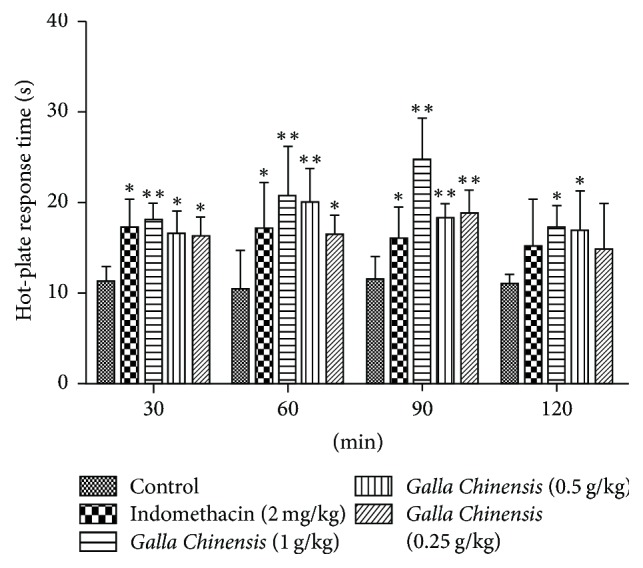
The electrical hot-plate study in mice. The values are presented as mean ± standard deviation (*n* = 10). The asterisk denotes the significance levels in comparison with the negative control: ^*∗*^*P* < 0.05; ^*∗∗*^*P* < 0.01.

As shown in Figure 1,* Galla Chinensis* (1 and 0.5 g/kg) significantly (*P* < 0.01) extended the threshold when compared to the control group at 60 min and 90 min after administration. Particularly, at 60 min after the administration, the time thresholds of* Galla Chinensis* groups were 24.50 ± 4.70 s, 18.05 ± 1.62 s, and 18.55 ± 2.66 s, respectively, which were significantly higher than that in control group (11.24  ±  2.62 s). The pain threshold in positive control group was also significantly (*P* < 0.05) increased at 30 min and 60 min after the administration.

### 3.2. Acetic Acid-Induced Writhing in Mice

The peripheral analgesic activities of* Galla Chinensis* on acetic acid-induced abdominal writhing in mice were shown in Table 1. Compared with the control group, the number of writhes in treated groups (*Galla Chinensis* and indomethacin) was significantly reduced (*P* < 0.01). The inhibition rate in the high, medium, and low doses of* Galla Chinensis*-treated groups reached 63.38%, 40.85%, and 38.03%, respectively. Indomethacin-treatment (2 mg/kg) also inhibited the writing (52.11%).

### 3.3. Xylene-Induced Ear Edema Test

The effects of* Galla Chinensis* on acute inflammation induced by xylene in mice were shown in Table 2. Compared with the control, the inhibition rates in the high (47.45%,* P* < 0.01) and medium (36.91%,* P* < 0.05) doses of* Galla Chinensis*-treated groups were significantly reduced. Meanwhile, indomethacin-treatment (2 mg/kg) inhibited ear edema with an inhibition rate of 50.12% (*P *< 0.01).

### 3.4. Acetic Acid-Indeed Capillary Permeability

**Figure 2:**
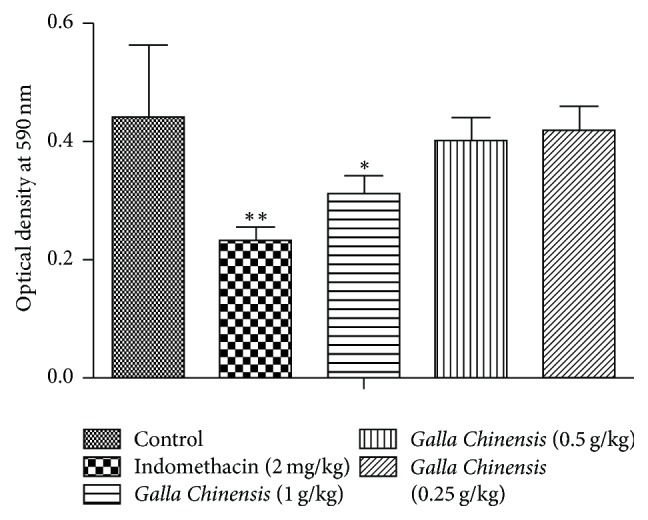
Effect of varying doses of* Galla Chinensis *on acetic acid-indeed capillary permeability. The values are presented as mean ± standard deviation (*n* = 10). The asterisk denotes the significance levels in comparison with the negative control: ^*∗*^*P* < 0.05; ^*∗∗*^*P* < 0.01.

As shown in Figure 2, compared with the control group, administration of* Galla Chinensis* (1, 0.5, and 0.25 g/kg) dose-dependently inhibited acetic acid-induced capillary permeability, and the high dose of* Galla Chinensis* had significant inhibitory effect (*P* < 0.05). Indomethacin markedly inhibited (*P* < 0.01) peritoneal capillary permeability induced by acetic acid.

### 3.5. Carrageenin-Induced Rat Paw Edema

The results were shown in Table 3. Compared with the control, the high dose of* Galla Chinensis* (1 g/kg) significantly reduced carrageenin-induced rat paw edema by 20.17% at 1 h and 27.19% at 2 h after administration (*P* < 0.05). The medium dose of* Galla Chinensis* (0.5 g/kg) could significantly inhibit edema by 29.04% (*P* < 0.05) at 2 h after administration. Meanwhile, the indomethacin (2 mg/kg) significantly decreased carrageenan-induced rat hind paw edema after administration.

**Figure 3:**
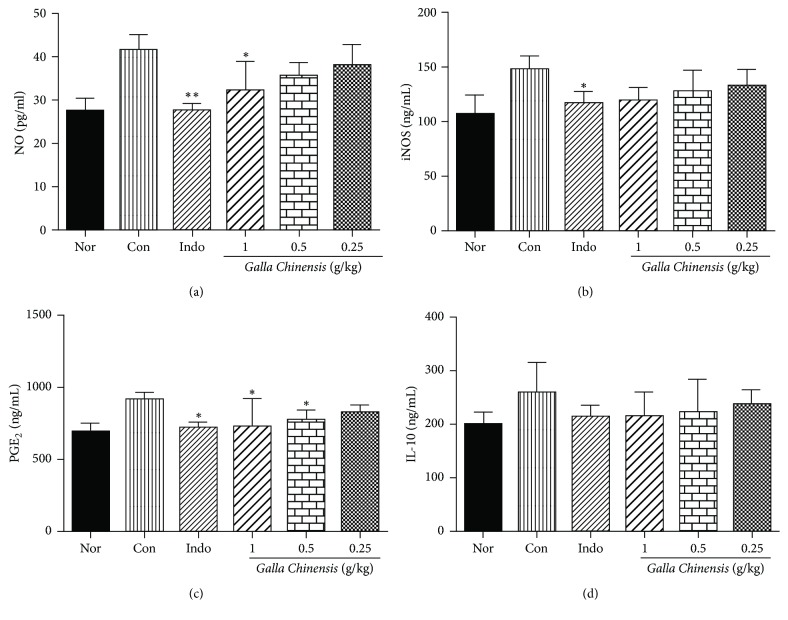
The changes of inflammatory cytokines in each group. The values are presented as mean ± standard deviation (*n* = 10). The asterisk denotes the significance levels in comparison with the control: ^*∗*^*P* < 0.05; ^*∗∗*^*P* < 0.01.

In order to estimate the analgesic and anti-inflammatory mechanisms of* Galla Chinensis*, several chemical mediators involving iNOS, NO, PGE_2_, and IL-10 were examined by ELISA. As shown in Figure 3, carrageenan-injection induced a remarkable increase in the concentrations of chemical mediators (iNOS, NO, PGE_2_, and IL-10) in serum. After treatment with* Galla Chinensis* (1 g/kg), the NO and PGE_2_ levels in serum were significantly reduced (*P* < 0.05) when compared with the control group.* Galla Chinensis* (0.5 g/kg) markedly decreased (*P* < 0.05) the PGE_2_ level. Indomethacin could significantly reduce the NO, iNOS, and PGE_2_ levels. No significant differences in IL-10 were observed between the treated groups and control group.

## 4. Discussion

It is well known that inflammation and pain are the most common diseases in human and animals, and the current treatment is to use steroidal and nonsteroidal anti-inflammatory drugs which have several side effects [20, 21].* Galla Chinensis*, a well-known traditional Chinese medicine, has a long history of being used for treating a lot of diseases. However, its analgesic and anti-inflammatory features have not been reported. In this study, we showed the potent analgesic and anti-inflammatory activities of* Galla Chinensis* in different animal models. The potency is almost equal to the positive drug (indomethacin).

The hot-plate test was used to evaluate central analgesic activity, and the peripheral analgesic activity was usually determined through acetic acid-induced writhing test [22]. In this study,* Galla Chinensis* and indomethacin could obviously increase the pain threshold of mice (Figure 1), indicating that* Galla Chinensis* had a potent central analgesic effect. Moreover, the number of writhes induced by acetic acid in* Galla Chinensis*-treated mice was significantly inhibited (Table 1), implying that* Galla Chinensis* had a commendable peripheral analgesic effect. In addition, the analgesic effect of* Galla Chinensis* showed a dose-dependent manner in the hot-plate test and acetic acid-induced writhing test.

The xylene-induced ear edema, acetic acid-indeed capillary permeability, and carrageenin-induced rat paw edema were the classic models for acute inflammation tests [23, 24]. In xylene-induced ear edema study,* Galla Chinensis* could significantly inhibit the ear edema and the inhibition rate in the high dose* Galla Chinensis*-treated group was equal to that in the indomethacin-treated group (Table 2), suggesting that* Galla Chinensis* possessed similar anti-inflammatory activity to indomethacin. Acetic acid has been widely used as a noxious agent to induce experimental inflammation [25]. In this study,* Galla Chinensis* could significantly inhibit peritoneal capillary permeability induced by acetic acid (Figure 2). In the carrageenin-induced rat paw edema study,* Galla Chinensis *(1 g/kg) significantly inhibited paw edema of rats in the early phase (within 120 min), especially the high dose of* Galla Chinensis*. These results indicated that* Galla Chinensis* possessed potent inhibitory effects against acute inflammation.

Inflammatory mediators are a kind of chemical medium with strongly biological activity, which can not only accommodate the release of it-self, but also activate other media systems that produce a series of cascade amplification reactions for further development of inflammation [26, 27]. It is reported that activated macrophages were irritated by carrageenan, which led to production of inflammatory mediators [28]. As shown in Figure 3, the contents of NO, iNOS, PGE_2_, and IL-10 were increased in carrageenan-treated groups, suggesting that carrageenan successfully caused inflammation and the inflammatory mediators (NO, iNOS, PGE_2_, and IL-10) were associated with the inflammatory process. NO is a short-lived small molecule which plays an important role in inflammatory condition. Harmful responses mediated excessive NO production, such as injury, septic shock, and apoptosis [29]. In carrageenan-evoked inflammation study, the contents of NO in the treated (*Galla Chinensis* and indomethacin) groups were significantly decreased. iNOS can produce excess NO against infections [30]. This study also showed that the levels of iNOS in the treated (*Galla Chinensis* and indomethacin) groups were decreased. These results suggested that the anti-inflammatory effects of* Galla Chinensis* and indomethacin may be attributed to reducing the content of iNOS which led to the reduction of NO.

Prostaglandin (especially PGE_2_) is important for inflammation diagnosis which is the main metabolite of arachidonic acid and is related to many pathophysiological processes, such as inflammation, tissue destruction, and tumor [31, 32]. This study showed that the contents of PGE_2_ in the treated (*Galla Chinensis* and indomethacin) groups were all obviously decreased (Figure 3(c)). These results suggested that the* Galla Chinensis* and indomethacin could slow down the inflammatory symptoms and had a remarkable inhibitory effect on the release of PGE_2_. Early results showed that IL-10 effectively inhibited the expression of cytokines and had an important role in stimulating immune and inflammatory responses [33]. Therefore, the level of IL-10 is also important for inflammation diagnosis. As shown in Figure 3(d), the contents of IL-10 in the treated (*Galla Chinensis* and indomethacin) groups were decreased, but no statistical differences were observed. These results suggested that* Galla Chinensis* had little influence on the production of IL-10.

## 5. Conclusions

*Galla Chinensis* has analgesic and anti-inflammatory activities. The possible anti-inflammatory mechanism is attributed to inhibition of the release of inflammatory cytokines and increasing the expression of anti-inflammatory factor. Hence,* Galla Chinensis* provides a good potential to be used to treat pain and inflammation.

## Figures and Tables

**Table 1:** Effects of *Galla Chinensis* on the acetic acid-induced writhing in mice.

Group	Dose	Writhing times	Inhibition (%)
Control	—	35.50 ± 1.91	—
Indomethacin	2 mg/kg	17.00 ± 3.83^*∗∗*^	52.11
*Galla Chinensis* high dose	1 g/kg	13.00 ± 3.37^*∗∗*^	63.38
*Galla Chinensis* medium dose	0.5 g/kg	21.00 ± 2.94^*∗∗*^	40.85
*Galla Chinensis* low dose	0.25 g/kg	22.00 ± 4.97^*∗∗*^	38.03

The values are presented as mean ± standard deviation (*n* = 10). The asterisk denotes the significance levels in comparison with the negative control: ^*∗∗*^*P* < 0.01.

**Table 2:** Effects of Galla Chinensis on xylene-induced ear edema test.

Group	Dose	Edema degree (mg)	Inhibition/%
Control	—	13.37 ± 3.37	—
Indomethacin	2 mg/kg	6.67 ± 2.50^*∗∗*^	50.12%
Galla Chinensis	1 g/kg	7.03 ± 1.23^*∗∗*^	47.45%
Galla Chinensis	0.5 g/kg	8.43 ± 2.72^*∗*^	36.91%
Galla Chinensis	0.25 g/kg	10.93 ± 2.29	18.21%

The values are presented as mean ± standard deviation (*n* = 10). The asterisk denotes the significance levels in comparison with the negative control: ^*∗*^*P* < 0.05; ^*∗∗*^*P* < 0.01.

**Table 3:** Effects of *Galla Chinensis* on carrageenan-induced rat paw edema.

Groups	Dose	Carrageenan-induced rat paw edema M ± SD (% inhibition of paw volume)					
		1 h	2 h	3 h	4 h	5 h	6 h
Control	—	35.83 ± 13.41	48.61 ± 10.29	46.39 ± 13.34	39.59 ± 7.45	39.56 ± 1078	35.82 ± 7.26
Indomethacin	2 mg/kg	22.88 ± 1.88^*∗*^ (36.14)	22.55 ± 7.55^*∗*^ (53.61)	24.45 ± 11.08^*∗*^ (47.29)	26.76 ± 10.37^*∗*^ (32.41)	22.88 ± 1.88^*∗*^ (42.16)	21.17 ± 10.45^*∗*^ (40.90)
*Galla Chinensis*	1 g/kg	20.17 ± 9.48^*∗*^ (43.71)	27.19 ± 5.69^*∗*^ (44.07)	29.41 ± 3.99 (36.60)	31.25 ± 8.20 (21.07)	30.19 ± 8.04 (23.69)	29.04 ± 6.48 (18.93)
*Galla Chinensis*	0.5 g/kg	25.96 ± 13.12 (27.55)	29.04 ± 6.48^*∗*^ (40.26)	30.13 ± 2.23 (35.05)	30.63 ± 7.14 (22.63)	31.17 ± 11.72 (21.21)	29.94 ± 10.32 (16.42)
*Galla Chinensis*	0.25 g/kg	33.38 ± 1.74 (6.84)	42.90 ± 13.49 (11.75)	39.88 ± 14.66 (14.03)	34.61 ± 8.24 (12.58)	34.51 ± 5.34 (12.77)	35.64 ± 7.61 (0.5)

The values are presented as mean ± standard deviation (*n* = 8). The asterisk denotes the significance levels in comparison with the negative control: ^*∗*^*P* < 0.05.
